# Fracture toughness of one- and two-dimensional nanoreinforced cement via scratch testing

**DOI:** 10.1098/rsta.2020.0288

**Published:** 2021-08-09

**Authors:** Ange-Therese Akono

**Affiliations:** Department of Civil and Environmental Engineering, Northwestern University, 2145 Sheridan Road, 60208 Evanston, IL, USA

**Keywords:** scratch tests, cement, graphene nanoplatelets, carbon nanotubes, carbon nanofibres, fracture toughness

## Abstract

Cement is the most widely consumed material globally, with the cement industry accounting for 8% of human-caused greenhouse gas emissions. Aiming for cement composites with a reduced carbon footprint, this study investigates the potential of nanomaterials to improve mechanical characteristics. An important question is to increase the fraction of carbon-based nanomaterials within cement matrices while controlling the microstructure and enhancing the mechanical performance. Specifically, this study investigates the fracture response of Portland cement reinforced with one- and two-dimensional carbon-based nanomaterials, such as carbon nanofibres, multiwalled carbon nanotubes, helical carbon nanotubes and graphene oxide nanoplatelets. Novel processing routes are shown to incorporate 0.1–0.5 wt% of nanomaterials into cement using a quadratic distribution of ultrasonic energy. Scratch testing is used to probe the fracture response by pushing a sphero-conical probe against the surface of the material under a linearly increasing vertical force. Fracture toughness is then computed using a nonlinear fracture mechanics model. Nanomaterials are shown to bridge nanoscale air voids, leading to pore refinement, and a decrease in the porosity and the water absorption. An improvement in fracture toughness is observed in cement nanocomposites, with a positive correlation between the fracture toughness and the mass fraction of nanofiller for graphene-reinforced cement. Moreover, for graphene-reinforced cement, the fracture toughness values are in the range of 0.701 to 0.717 MPa$\sqrt{\text{m}}$. Thus, this study illustrates the potential of nanomaterials to toughen cement while improving the microstructure and water resistance properties.

This article is part of a discussion meeting issue ‘A cracking approach to inventing new tough materials: fracture stranger than friction’.

## 1. Introduction

Concrete is the second most-consumed resource on Earth after water, with a global production that exceeds 16 billion metric tonnes a year [1,2]. Cement is an essential ingredient in concrete, with an annual world production of 4.1 billion metric tonnes [3]. However, the production of a tonne of cement releases a tonne of carbon dioxide into the atmosphere. As a result, the cement industry accounts for 8% of global human-caused carbon dioxide emissions [4]. Thus, the carbon footprint of the cement industry must be cut drastically to meet the Paris Agreement's temperature goals.

A significant way to decrease the carbon footprint of cement is to develop cements with improved fracture resistance to reduce the volume needed to design buildings and structures. In recent years, carbon-based nanomaterials have attracted much attention as a potential reinforcement for cement due to their extraordinary properties. For instance, carbon nanotubes exhibit a tensile strength of 5–100 GPa and Young's modulus of 1 TPa [5–9]. Meanwhile, graphene exhibits a tensile strength of 130 GPa and Young's modulus of 1 TPa [10]. Several studies have reported significant gains in stiffness and strength using cement nanocomposites reinforced with carbon-based nanomaterials [11–14]. The improvement in mechanical properties is usually followed by an enhancement in multifunctional response, such as electrical conductivity or strain-sensing capabilities. In particular, a critical issue has been to increase the fraction of carbon-based nanomaterials—to maximize the multifunctional behaviour—while controlling the microstructure and improving the mechanical characteristics. However, when it comes to mechanical performance, the primary criterion has been strength. The issue is that a strength-focused performance criterion cannot account for defects, which are pervasive at the structural level, nor can it account for fracture, which plays an important role in the failure. Thus, more studies are needed to yield advanced synthesis protocols for the synthesis of Portland cement carbon-based nanocomposites, and understand the influence of carbon-based nanomaterials on the fracture response of Portland cement.

The objective of this research is to employ novel fracture assessment methods and novel synthesis protocols to study the impact of carbon-based nanomaterials on the fracture of cement reinforced with nanomaterials. The focus is on graphene nanoplatelets, carbon nanofibres, multiwalled carbon nanotubes and helical carbon nanotubes. To this end, scratch testing is employed, which consists of pushing a sphero-conical probe across the surface of the specimen. This manuscript is organized as follows: First, the experimental programme is introduced. Then, the theoretical model is presented. Finally, the results are shown, and their significance is analysed.

## 2. Materials and methods

### (a) Cement nanocomposite design and synthesis

Cement composites reinforced with carbon-based nanofillers were synthesized. Four types of carbon-based nanofillers were selected: carbon nanofibres (CNF), multiwalled carbon nanotubes (MWCNT), helical carbon nanotubes (HX) and graphene nanoplatelets (GNP). The carbon nanofibres were sourced from Pyrograph Products, Inc. (Cedarville, OH) as highly graphitic and tubular stacked-cup carbon nanotubes. The remaining carbon-based nanofillers were sourced from Cheap Tubes, Inc. (Grafton, VT). Both multiwalled carbon nanotubes and helical carbon nanotubes were produced through catalytic chemical vapour deposition. The helical carbon nanotubes contained 80 wt% carbon nanotubes with a helical structure and various helix angles. Furthermore, chemically exfoliated graphene nanoplatelets were also considered, with a thickness of 8–15 nm. The specific surface area was highest for the graphene nanoplatelets (500–700 m^2 ^g^−1^) and lowest for the carbon nanofibres (20–30 m^2 ^g^−1^). Meanwhile, the reverse was true for the nanofiller length: the highest value was achieved with carbon nanofibres (150–200 µm), whereas the lowest was achieved with graphene nanoplatelets (1–2 µm). Table S1 in the electronic supplementary material section lists the specific surface area, diameter and length of each carbon-based nanofiller considered.

Cement nanocomposites were synthesized, through a novel procedure, with 0.1–0.5 wt% carbon-based nanofillers per mass of cement. The detailed mix designs of all cement nanocomposites are provided in table S2 of the electronic supplementary material. In the rest of the manuscript, we will designate the specimens as *X*-*n* where *X *= (GNP, CNF, HX and MWCNT) is the type of nanomaterial and *n* = (1, 2, 5) represents the mass fraction of nanomaterial (0.1*n wt% per mass of cement).

The novel synthesis protocol involved four steps. First, the carbon-based nanofillers were pre-dispersed in deionized water with ultrasonic energy. A quadratic distribution of ultrasonic energy was adopted with respect to the nanofiller mass fraction. The amount of ultrasonic energy provided was 1.77 kJ/(g l^−1^) for 0.1 wt%, 3.54 kJ/(g l^−1^) for 0.2 wt% and 17.72 kJ/(g l^−1^) for 0.5 wt%. Second, the suspension of carbon-based nanofillers in deionized water was mixed with Portland cement using an overhead IKA digital stirrer equipped with a four-bladed propeller stirrer to provide ultra-high speed and high shear. The mixing speed was set at 200 rpm for nanofiller fractions less than 0.1 wt%, 400 rpm for 0.2 wt%, and 800 rpm for 0.5 wt%. Afterwards, the slurry was poured into lubricated cylindrical moulds that were then sealed using polyethylene films. The specimens were initially cured for 24 h using an orbital shaker at a rotational speed of 79 rpm. After the initial 24 h curing, the cement nanocomposite specimens were removed from their moulds and soaked in deionized water for an additional 6 days. After a total of 7 days of curing, the cement nanocomposites were soaked in ethanol for 24 h to stop the cement hydration and stored under vacuum afterwards.

Two reference Portland cement materials were mixed by combining 138.8 g of Portland cement with 61.12 g of deionized water. For the first reference Portland cement specimens, R-M, the Portland cement powder and deionized water were mixed manually for 2 min and cast in lubricated, sealed moulds to cure at room temperature for 24 h. For the second reference Portland cement specimens, R-HS-OS, the cement powder was mixed with deionized water using an IKA digital overhead, high-shear, high-speed mixer at 200 rpm for 2 minutes. Afterwards, the slurry was cast in lubricated moulds and sealed using an orbital shaker with a 19 mm orbit and rotational speed of 79 rpm for 24 h. For both reference cement materials, R-M and R-HS-OS, after 24 h of curing, the cement specimens were removed from their moulds and cured in deionized water for 7 days.

### (b) Water absorption and porosity measurements

Water absorption and porosity were measured after 7 days of curing following standard ASTM C20-00 [15] with minor modifications. First, the specimens were dried in an oven at 50°C for 24 h, and the dry mass *M*_dry_ and dry specific gravity ${\text{ρ}}_{\text{dry}}$ were measured. Then, the specimens were saturated by submersion in deionized water at 23°C for 24 h, and the saturated mass $M_{\text{saturated}}$ was measured. The water absorption *W* was calculated as the relative difference between the dry and the saturated mass:

2.1 $$W=\frac{M_{\text{saturated}}-M_{\text{dry}}}{M_{\text{dry}}}\times 100.$$

The porosity *P* was computed as the product of the water absorption and the dry specific gravity [16]:

2.2 $$P=W\times \rho_{\text{dry}}.$$

### (c) Grinding and polishing

Before nanoscale mechanical testing, the cement nanocomposite specimens were meticulously polished to yield a flat surface. First, each specimen was cold-mounted using a low-viscosity epoxy resin. Afterwards, 4 mm thick slices were machined using a low-speed diamond saw with an inert, oil-based coolant. Grinding was conducted using a semi-automated grinder and polisher apparatus, along with silicon carbide grinding pads of grit size 240, 400 and 600, consecutively. The specimens were rinsed using an ultrasonic bath with an inert, oil-based solvent in between each grit size. Polishing was conducted using abrasive lapping discs with silicon carbide particles of size 1 µm and 0.25 µm, consecutively. After grinding and polishing, the specimens were stored under a vacuum.

### (d) Environmental scanning electron microscopy imaging

The microstructure of the polished cement nanocomposite specimens was observed using environmental scanning electron microscopy (ESEM) imaging. To this end, an FEI Quanta 650 environmental scanning electron microscope equipped with a backscatter detector was used. In the ESEM experiments, the walking distance was 10–11 mm, the accelerating voltage was 10 kV, the spot size was 3–4.5 and the magnification level was in the range of 10 000× to 50 000×.

### (e) Scratch tests

**Figure 1.**
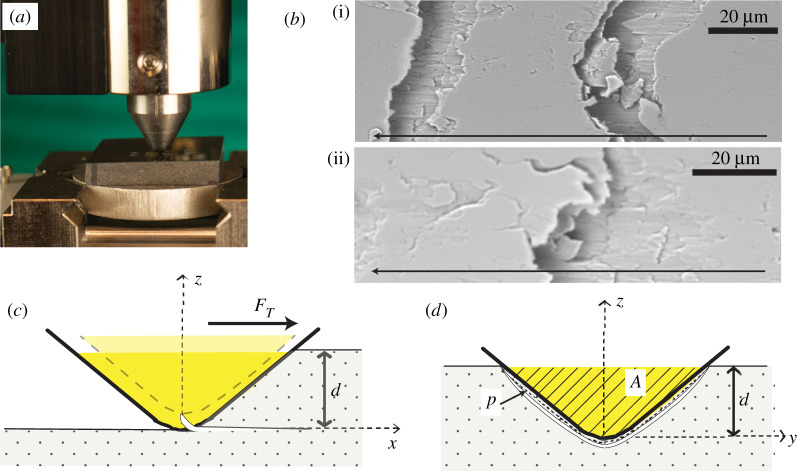
(*a*) Digital photography image of scratch test equipment set-up. Credits: Ange-Therese Akono, Northwestern University, 2020. (*b*) Backscattered environmental scanning electron micrographs of the residual groove after progressive-load scratch testing on cold-rolled steel. The arrows indicate the direction of the scratch test. (*c,d*) Schematics illustrating the Fracture Mechanics model for scratch tests using an axisymmetric probe. (*c*) Side view. (*d*) Front view. The hatched area *A* is the horizontally projected load-bearing contact area. The dotted red line *p* is the perimeter. *F_T_* is the horizontal force and *d* is the penetration depth. Adopted from Akono *et al*. [17], with permission from Cambridge University Press. (Online version in colour.)

The fracture response of the cement nanocomposite specimens was measured using microscopic scratch tests. As illustrated in figure 1*a*, scratch tests consisted of pushing an axisymmetric probe across the surface of a softer material under a linearly prescribed vertical force. All scratch tests were conducted using an Anton Paar (Ashland, VA) microscopic scratch tester equipped with a 200 µm Rockwell C diamond probe. The vertical force was progressively increased using a force feedback loop system, and the prescribed maximum vertical force was 2.5 N. Meanwhile, the scratch length was 5 mm, and the scratch speed was 10 mm/min. Before testing the cement specimens, the scratch probe was calibrated using fused silica. Calibration scratch tests were performed with a maximum vertical force of 7 N, a scratch length of 3 mm and a scratch speed of 6 mm min^−1^. During each scratch test, the vertical and horizontal forces were measured using load sensors with a resolution of 0.1 mN. The penetration depth was measured using a linear variable differential transformer system with a resolution of 0.3 nm. The acquisition rate for the forces and the vertical depth was 45.0 kHz. For each cement nanocomposite material, seven scratch tests were conducted, spaced 1.2 mm apart. The microscopic scratch tester unit was integrated with a high-resolution Nikon transmitted light microscope. At the end of each scratch test, optical microscopy images of the residual top surface were captured using an Olympus objective at 200 × magnification, yielding a scratch panorama. After scratch testing, fracture micromechanisms were investigated using backscattered ESEM.

## 3. Theory

A nonlinear fracture mechanics model was applied to extract the fracture toughness from the scratch test measurements. The theoretical model was previously published in [18–20]; the salient points are summarized here. The first step is to identify the shape of the fracture surface during scratch testing. To this end, backscattered ESEM imaging was performed to visualize the residual groove following progressive-load scratch tests on cold-rolled steel with a sphero-conical probe (figure 1*b*). Curved fracture surfaces were observed perpendicular to the direction of scratch testing. As for the orientation, the fracture surfaces were slanted, suggesting subsurface cracking. Based on these observations, the existence of a crack that propagates forward beneath the surface, away from the tip of the scratch probe, was postulated (figure 1*c*).

**Figure 2.**
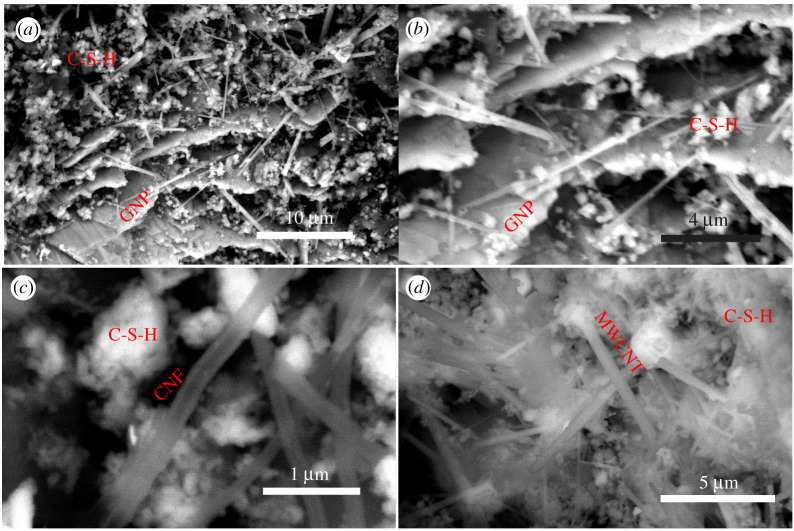
Backscattered environmental scanning electron microscopy of plain cement and cement nanocomposites. Unless otherwise noted, all specimens were imaged after 7 days of hydration. (*a,b*) Graphene-reinforced cement, GNP-5. (*c*) Carbon nanofibre–reinforced cement, CNF-5. (*d*) Multiwalled carbon nanotube–reinforced cement, MWCNT-5. C-S-H = calcium silicate hydrates. GNP = graphene nanoplatelets. CNF = carbon nanofibre. MWCNT = multiwalled carbon nanotubes. (Online version in colour.)

As drawn in figure 1*d*), the crack initiated below the crack tips and later followed the contour of the scratch probe. Consider a crack of length ℓ that propagates at a speed $\underline{V}=\dot{\ell }{\underline{e}}_{x}$. Given penetration depth *d*, there are two geometrical parameters of interest: the perimeter *p* (the dotted line in figure 2*b*) and the horizontally projected load-bearing contact area *A* (the hatched area in figure 1*c*). During an incremental advance dℓ of the crack, the incremental crack surface created is dΓ=pdℓ. The energy release rate *G* is the thermodynamic driving force associated with crack propagation. The dissipation during crack propagation is dD=GdΓ. The energy release *G* is then related to the rate of change of the potential energy $E_{\text{pot}}$ via:

3.1 $$\frac{\text{d}E_{\text{pot}}}{\text{d}t}=-G\dot{\Gamma }=-Gp\dot{\ell }.$$

The energy release rate *G* is calculated using the contour integral method or *J*-integral [21]. The basic idea is to describe the change in potential energy within a material volume Ω from the perspective of an observer tied to the tip of the propagating crack. Consider a closed contour *C* containing the crack tip. For a displacement-prescribed test, and given the stress-free boundary conditions on the crack faces, the potential energy is the integral of the free energy ψ inside the material volume Ω, or $E_{\text{pot}}=\int_{\Omega }\psi d\Omega .$ The total change in potential energy comprises two terms. The first term, $\int_{\Omega }(\partial \psi \text{/}\partial t)d\Omega$, is related to the change in free energy within Ω. The second term, $\int_{\partial \Omega }-\psi \underline{V}\cdot \underline{n}$, describes the free energy convectively transported as the reference system moves at speed $\underline{V}$ (the observer is fixed and tied to the crack tip) where $\underline{n}$ is the outward unit vector normal to the boundary ∂Ω of Ω. Thus:

3.2 $$\frac{\text{d}E_{\text{pot}}}{\text{d}t}=\int_{\Omega }\frac{\partial \psi }{\partial t}\;\text{d}\Omega -\int_{\partial \Omega }\psi \underline{V}\cdot \underline{n}=-Gp\dot{\ell }.$$

For a linear elastic material, $\psi =(1/2)\underline{\underline{\sigma }}:\underline{\underline{\varepsilon }}$. Using the theorem of virtual work, the first term can be transformed into $\int_{\Omega }\left(\partial \psi \text{/}\partial t\right)\;\text{d}\Omega =\dot{\ell }\int_{\partial \Omega }\underline{T}\cdot (\partial \underline{\xi }\text{/}\partial x)\text{d}S,$ where $\underline{T}=\underline{\underline{\sigma }}\cdot \underline{n}$ is the stress vector on the boundary ∂Ω and $\underline{\xi }$ is the displacement vector. Finally, the integral on the boundary of the material volume element can be simplified into an integral on the closed contour *C*, since the crack surface is stress-free $\left(\underline{T}=\underline{0}\right)$ and horizontal $\left({\underline{e}}_{x}\cdot \underline{n}=0\right)$. Therefore, the energy release rate can be estimated from:

3.3 $$G=\frac{1}{p}\int_{C}\left(\psi n_{x}-\underline{T}\cdot \frac{\partial \underline{\xi }}{\partial x}\right)\text{d}S.$$

with $n_{x}={\underline{e}}_{x}\cdot \underline{n}$. In the case of the scratch test, the closed contour comprises the material probe interface (S), the top surface that is stress-free $\left(n_{x}=0,\underline{T}=\underline{0}\right)$, and closing material surfaces far removed $\left(\psi =0,(\partial \underline{\xi }/\partial x)=\underline{0}\right)$. As a result, the only non-zero contribution to the right-hand side of equation (3.1) comes from the material–probe interface:

3.4 $$G=\frac{1}{p}\int_{\left(S\right)}\left(\psi n_{x}-\underline{T}\cdot \frac{\partial \underline{\xi }}{\partial x}\right)\text{d}S.$$

Assuming plane strain conditions, along with a uniaxial distribution of the stress field ahead of the probe, $\underline{\underline{\sigma }}=-(F_{T}\text{/}A){\underline{e}}_{x}⊗{\underline{e}}_{x},$ we can express the energy release rate *G* as a function of the horizontal force $F_{T}$, the material Young's modulus *E* and the Poisson's ratio *v* according to:

3.5 $$G=\frac{1-\upsilon^{2}}{E}\frac{F_{T}^{2}}{2pA}.$$

The Griffith crack propagation criterion is employed to mark the onset of crack propagation. The crack propagates when the energy release rate *G* reaches a certain threshold, that is, the fracture energy $G_{f}$ [22]. The Griffith–Irwin relation is used to connect the fracture energy $G_{f}$ to the fracture toughness $K_{c}$, assuming plane strain conditions $G=((1-\upsilon^{2})/E)K_{c}^{2}$ [23]. The fracture toughness $K_{c}$ is then a function of the horizontal force $F_{T}$ according to:

3.6 $$K_{c}=\frac{F_{T}}{\sqrt{2pA}}.$$

Herein, 2*pA* is the scratch probe shape function that depends on the penetration depth *d* and on the scratch probe geometry. For instance, for a conical probe, the scratch probe shape function is a cubic function of the penetration depth, whereas for a spherical probe, the scratch probe shape function is a quadratic function of the penetration depth. In practice, the scratch probe shape function must be calibrated using a reference material [24]. The theoretical model then predicts that, in the case of a purely brittle fracture process, the ratio of the horizontal force over the square root of the scratch probe shape function is constant and equal to the fracture toughness of the material.

## 4. Results

### (a) Microstructure of cement nanocomposites

Using ESEM, a granular and porous microstructure was observed, with unhydrated cement grains in white, hydrated cement in grey and micropores in black (electronic supplementary material, figure S2). Figure 2 displays high-resolution BESEM images of nano-reinforced cement at a mass fraction of 0.5 wt%, with magnification levels ranging between 5000 × and 50 671 ×. Additional BESEM images are shown in electronic supplementary material, figure S3. For graphene-reinforced cement (figure 2*a,b*), flakes of graphene nanoplatelets were observed connecting cement hydration products—here, calcium silicate hydrate (C-S-H) grains and ettringite needles. For carbon nanofibre-reinforced cement, figure 2*c* shows single carbon nanofibres, 110–240 nm thick, filling nanopores. BESEM imaging suggests that the dispersion procedure was sufficient to debulk carbon nanofibres and yield isolated carbon nanofibres within Portland cement matrices. As for multiwalled carbon nanotube–reinforced cement, figure 2*d* shows carbon nanotube bundles, 145–365 nm thick, filling nanopores and connecting C-S-H grains. Thus, nanomaterials refined the pore structure at the nanoscale by filling voids and connecting cement hydration products. These findings agree with recent studies that reported a refinement of the pore size in cement nanocomposites [14,25–27].

Our novel procedure for synthesizing cement nanocomposites yielded an increase in water penetration resistance, as measured via water absorption and porosity. The water absorption and porosity values for the reference cement and cement nanocomposite specimens are reported in electronic supplementary material, figures S4*a* and S4*b*, respectively. The nominal values of water absorption, porosity and dry specific gravity are given in electronic supplementary material, table S3. The water absorption of the manually mixed Portland cement reference specimen was 16.83%, with a porosity of 29.76%. Significant decreases in both water absorption and porosity were observed for the Portland cement reference specimen following high-speed, high-shear mixing combined with curing on an orbital shaker: water absorption was 5.07% and porosity was 9.64%. A greater decrease was observed for cement nanocomposites, with median water absorption and porosity values of 3.65% and 7.17%, respectively. The lowest water absorption and porosity values were obtained for GNP-5: 2.52% and 4.81%, respectively. These findings agree with Hu *et al*.'s study [25]. After testing cement reinforced with 0.05–0.1 wt% carbon nanotubes, the volume fraction of pores greater than 100 nm was 3–5%. Therefore, 24 h curing on an orbital shaker was essential to remove macroscopic air voids, densify the microstructure, and reduce water absorption. Carbon-based nanomaterials also promoted the densification of the microstructure and led to an improvement in water penetration resistance, as measured by water absorption and porosity.

### (b) Fracture micromechanisms of cement nanocomposites

**Figure 3.**
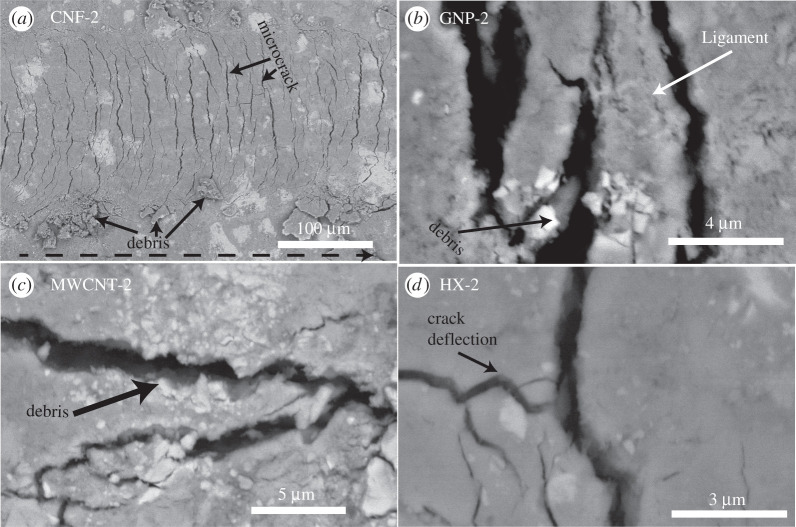
Fracture mechanisms of cement nanocomposites. (*a*) Carbon nanofibre–reinforced cement (CNF-2). The dotted arrow indicates the direction of the motion of the scratch probe. (*b*) Graphene-reinforced cement (GNP-2). (*c*) Multiwalled carbon nanotube–reinforced cement (MWCNT-2). (*d*) Helical carbon nanotube–reinforced cement (HX-2).

Figure 3*a* displays a BESEM image of the residual groove after scratch testing on carbon nanofibre–reinforced cement. Microcracks were observed that were curved and perpendicular to the direction of the motion of the scratch probe. There was also some debris present on the sides of the grooves. The microcracks and debris provided physical evidence of fracture processes during scratch testing and justified the use of the scratch test method to yield fracture toughness. Figure 3*b***–***d* display fracture micromechanisms for cement nanocomposites. In addition to microcracking and debris generation, additional fracture micromechanisms include ligament bridging and crack deflection.

### (c) Fracture toughness of cement nanocomposites

**Figure 4.**
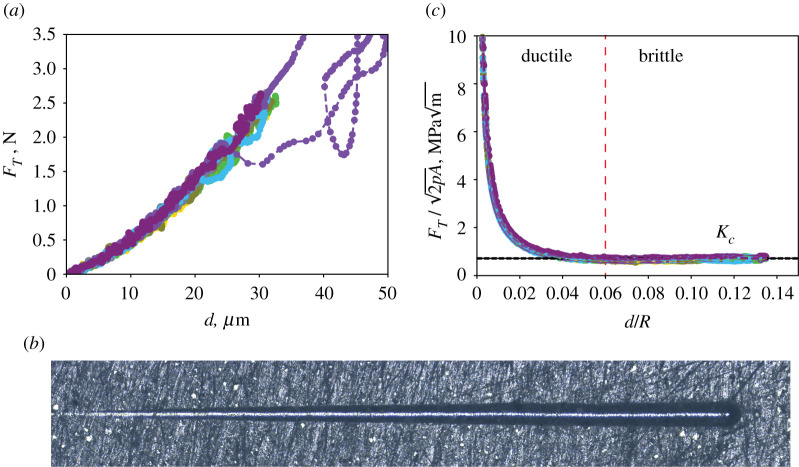
Fracture toughness of graphene–cement GNP-5. (*a*) Load-depth curve. (*b*) Residual groove after scratch testing. (*c*) Fracture scaling curve. *F_T_* is the horizontal force, *d* is the penetration depth, *R* is the scratch probe tip radius and 2*pA* is the scratch probe shape function. *K_c_* is the fracture toughness. (Online version in colour.)

**Figure 5.**
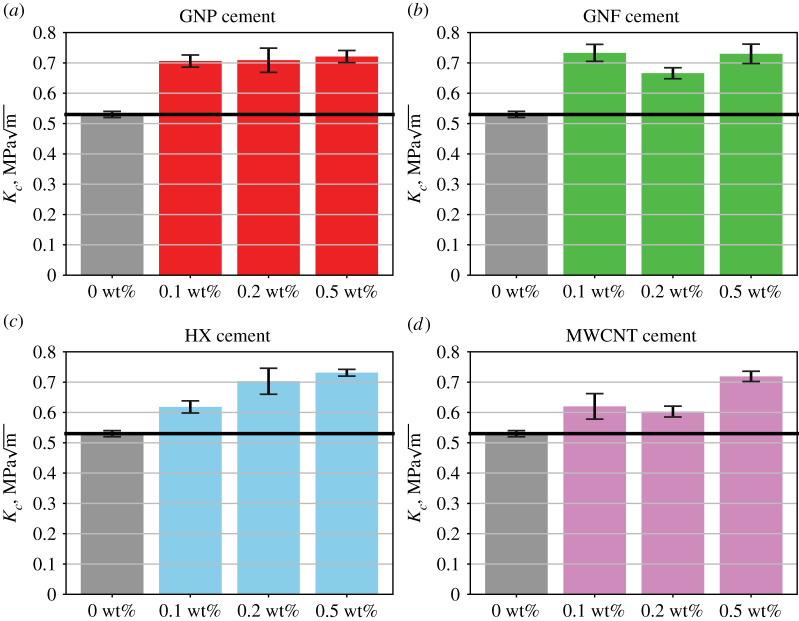
Influence of carbon-based nanomaterials on the fracture toughness of cement matrices. GNP = graphene nanoplatelets. CNF = carbon nanofibres. HX = helical carbon nanotubes. MWCNT = multiwalled carbon nanotubes. The solid blue line indicates the average fracture toughness of the reference Portland cement specimen. (Online version in colour.)

Figure 4*a* displays representative load-depth curves for cement nanocomposites, using graphene cement GNP-5 as an example. Seven scratch tests were conducted, spaced 1.2 mm apart. All seven tests look similar when superimposed on top of each other, pointing to the reproducibility of the scratch test method. For all but one test, the maximum penetration depth was approximately 32 µm, whereas the maximum value of the horizontal force was around 2.5 N. After each scratch test, a residual groove was formed (figure 4*b*). The presence of the residual groove, along with the observed crack surfaces shown in figure 5, support our approach to measuring fracture toughness using scratch testing. Supplementary figure 4 displays the load-depth curves for all cement nanocomposite specimens tested.

**Figure 6.**
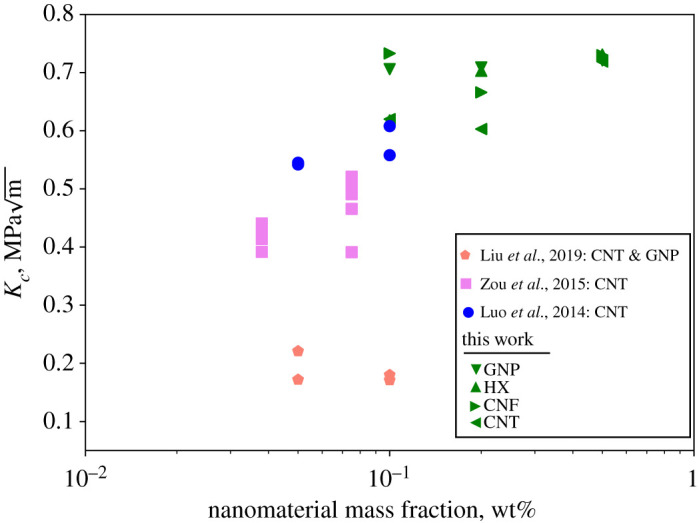
Comparison of the findings in this study to recent works on cement reinforced with carbon-based nanomaterials. GNP = graphene nanoplatelets. CNF = carbon nanofibres. HX = helical carbon nanotubes. CNT = carbon nanotubes. (Online version in colour.)

Figure 4*c* displays the fracture scaling of scratch tests using the nonlinear fracture mechanics model. The quantity $F_{T}\text{/}\sqrt{2pA}$ is displayed as a function of the depth-to-radius ratio *d*/*R*. A Rockwell C scratch probe was used, which consists of a cone of half-apex angle 60° with a sphere of tip radius R=200 μm at the end. In particular, the transition from sphere to cone occurs at a penetration depth of $d_{0}=27\;\text{μ}\text{m}$. Thus, given the penetration depths recorded (figure 6*b*), we used the shape function for a spherical probe and considered only data points corresponding to $d\leq d_{0}$. According to the theoretical model (equation (3.4)), the quantity $F_{T}\text{/}\sqrt{2pA}$ is constant in the case of a brittle fracture process. Figure 4*c* shows two regions: for d/R≤0.06, the quantity $F_{T}\text{/}\sqrt{2pA}$ decreases sharply, pointing to a mix of ductile and brittle failure processes. However, for *d*/*R *> 0.06, a convergence towards a constant value is observed. The convergence of $F_{T}\text{/}\sqrt{2pA}$ points to a fracture-driven process with dominant brittle fracture; fracture toughness is also the asymptotic value of $F_{T}\text{/}\sqrt{2pA}$. Before the test, the scratch probe shape function 2*pA* was calibrated using fused silica [19]; the calibration curve is shown in electronic supplementary material figure S5. Using scratch tests, the fracture toughness of the reference Portland cement specimen R-M was found to be equal to $0.531\pm 0.006\text{M}Pa\sqrt{\text{m}}$. This value agrees with reported values of the fracture toughness for plain Portland cement (w/c = 0.4, 0.4–0.5 MPa$\sqrt{\text{m}}$) using conventional macroscopic fracture testing methods, such as the three-point bending test on single-edge notched specimens [28–30]. This agreement in fracture toughness measurement between the scratch test fracture approach and conventional fracture testing method for plain Portland cement supports the rigour and validity of our approach. Electronic supplementary material, figure S7 displays the load-depth curves and fracture toughness scaling curves for all Portland cement specimens. The high-shear, high-speed mixing and the curing with an orbital shaker were found to significantly enhance fracture resistance, as the reference Portland cement R-HS-OS exhibited a 26% increase in fracture toughness, with a fracture toughness value of $0.67\pm 0.02\text{M}Pa\sqrt{\text{m}}$, consistent with the reduction of porosity due to the improved mixing/casting method.

Figure 5 displays fracture toughness values measured via scratch tests for all cement nanocomposites considered in this study. Electronic supplementary material, figure S8 displays the fracture scaling curves, and electronic supplementary material, table S4 in the electronic supplementary material lists the fracture toughness values for all 12 cement nanocomposites. For graphene-reinforced cement, the fracture toughness ranged from 0.706 to 0.721 MPa$\sqrt{\text{m}}$. Moreover, a positive correlation was observed between the mass fraction of graphene nanoplatelets and the gain in fracture toughness. For carbon nanofibre–reinforced cement, the fracture toughness ranged from 0.666 to 0.733 MPa$\sqrt{\text{m}}$. For cement reinforced by multiwalled carbon nanotubes and helical carbon nanotubes, the fracture toughness ranged from 0.603 to 0.719 MPa$\sqrt{\text{m}}$ and 0.618 to 0.731 MPa$\sqrt{\text{m}}$, respectively. Graphene nanoplatelets exhibited the highest specific surface area, 500–700 m^2 ^g^−1^, which could explain the higher values of the fracture toughness as the mass fraction of graphene nanoplatelets increased. Similarly, carbon nanofibres exhibited the highest length, 50–200 µm, which could explain the enhancement in fracture toughness for all three reinforcement levels. Thus, our study suggests that the specific surface area and the length of nanomaterials play a significant role in regulating the fracture toughness of cement nanocomposites.

## 5. Discussion

A new method is shown to disperse high-mass fractions of carbon-based nanomaterials in Portland cement matrices while enhancing fracture toughness. Due to strong Van der Waals forces, nanomaterials such as carbon nanotubes, carbon nanofibres and graphene nanoplatelets tend to agglomerate and form clusters. Nanomaterial clusters, in turn, hinder the workability of the cement slurry, promote the formation of macropores and limit load transfer mechanisms within the hardened cement nanocomposite. The challenge then consists of dispersing the nanomaterials within the cement matrix while enhancing mechanical performance. The standard is to disperse nanomaterials using ultrasonic energy combined with an air-entraining agent, such as a carboxylate-based superplasticizer [31–33]. The ultrasonic energy provided is also typically linearly proportional to the fraction of nanomaterials [34]. By contrast, in this study, nanomaterials are dispersed in three steps: pre-dispersion in deionized water using ultrasonic energy, mixing with cement using ultrahigh-speed, high-shear conditions, and continuous mechanical stirring for the first 24 h of curing using an orbital shaker. The ultrasonic energy scales quadratically with respect to the mass fraction of nanomaterials. In the last two steps, unhydrated cement grains are used to further disperse nanomaterial clusters. Moreover, the role of ultrahigh-speed, high-shear conditions is to promote nanomaterial cluster dispersion and accelerate cement hydration. Finally, in the first 24 h of the curing process, an orbital shaker is used to remove macroscopic air voids.

The novel synthesis route presented in this study yielded increases in the fracture toughness of cement nanocomposites. Figure 6 compares our findings to recent studies that have measured the fracture toughness of Portland cement paste reinforced with carbon-based nanomaterials, such as carbon nanotubes and graphene nanoplatelets. Luo *et al*. [35] measured the fracture toughness of CNT-reinforced cement using three-point bending tests on single-edge notched specimens. They reported fracture toughness values ranging from 0.1757 to 0.3242 MPa$\sqrt{\text{m}}$ for 0.1–0.2 wt% CNT-reinforced cement after 28 days of curing. Hu *et al*. [25] measured the fracture toughness of CNT with w/c = 0.2 after 48 h of curing. The fracture toughness was 0.542–0.608 MPa$\sqrt{\text{m}}$ for 0.05–0.1 wt% CNT-reinforced cement. Zou *et al*. [30] investigated the fracture resistance of CNT cement with w/c = 0.4 at mass fractions 0.038% and 0.075% for various values of ultrasonic dispersion energy and after 28 days of curing. However, they concluded that the optimal dispersion energy was constant, irrespective of the CNT mass fraction. The fracture toughness values for the optimal mix design were 0.408 MPa$\sqrt{\text{m}}$ for 0.038% CNT and 0.506 MPa$\sqrt{\text{m}}$ for 0.075% CNT. Finally, Liu *et al*. [26] studied the fracture behaviour of CNT cement and GNP cement with w/c = 0.35 and mass fractions of 0.05 wt% and 0.1 wt% after 28 days of curing. The fracture toughness was 0.180–0.221 MPa$\sqrt{\text{m}}$ for CNT cement and 0.171–0.172 MPa$\sqrt{\text{m}}$ for GNP cement. The fracture toughness decreased as the mass fraction of nanomaterials increased.

It is challenging to compare the fracture toughness of cement nanocomposites between studies due to differences in the type, geometry and source of nanomaterials. One must also account for differences in cement nanocomposite w/c ratios, curing age and curing regimes. Another factor is the length-scale of testing. For instance, macroscopic specimens might involve a higher distribution of defects that would result in lower fracture toughness. However, the methodology presented in this study yields both higher reinforcement levels and higher fracture toughness values for carbon nanotube–reinforced cement, carbon nanofibre–reinforced cement and graphene-reinforced cement. After 7 days of curing, for w/c = 0.44, and for reinforcement mass fractions of 0.1–0.5 wt%, the lowest fracture toughness value was 0.599 MPa$\sqrt{\text{m}}$ and the highest value was 0.726 MPa$\sqrt{\text{m}}$. Some areas for future investigation include the effect of curing conditions and curing age on the fracture toughness of cement nanocomposites. Nevertheless, a novel synthesis route has been devised to yield cement nanocomposites with high reinforcement levels of carbon-based nanomaterials and with significantly improved values of fracture toughness.

This study has demonstrated the potential of scratch testing to yield the fracture toughness of cement nanocomposites. The observed fracture-enhancing mechanisms were pore refinement, microcracking, crack deflection, ligament bridging and debris formation. Additional fracture micromechanisms reported in the scientific literature for nano-reinforced cement involve nanomaterials bridging microcracks [25,26,35] and nanomaterial pulling out [30,36]. The advantage of the scratch test method is that it is semi-destructive, reproducible and requires small specimens. The specimens tested in this study were 30 mm wide and 3 mm thick. By contrast, conventional fracture testing methods, such as the three-point bending test, require macroscopic specimens—usually 40 × 40 × 160 mm—along with fastidious specimen preparation. Furthermore, it is essential to generate a sharp notch to yield an accurate fracture toughness measurement in the three-point bending method. In practice, a finite notch radius is used, which can result in significant measurement inaccuracies [37,38]. Another issue is the presence of significant size effects due to the interaction between the fracture process zone and the specimen dimensions [39]. The scratch test, on the other hand, does not require an initial notch to be created. Moreover, size-independent fracture toughness is obtained in the asymptotic regime of brittle fracture. Thus, the scratch test provides an alternative means to probe fracture toughness at the microscopic length-scale using depth-based sensing techniques.

## 6. Conclusion

We investigated the influence of carbon-based nanomaterials on the fracture response of Portland cement nanocomposites. We devised novel synthesis routes to incorporate 0.1–0.5 wt% graphene nanoplatelets, helical carbon nanotubes, multiwalled carbon nanotubes and carbon nanofibres into cement matrices. Here are our major findings:

— Graphene nanoplatelets exhibited two distinct morphologies in graphene-reinforced cement: open flakes and rolled-up tubes. Due to their large specific surface area, there is a positive correlation between the fraction of graphene nanoplatelets and the fracture toughness of the resulting nanocomposites. The fracture toughness of graphene-reinforced cement ranged from 0.706 to 0.721 MPa$\sqrt{\text{m}}$.
— For helical carbon nanotubes, multiwalled carbon nanotubes and carbon nanofibre–reinforced cement, an increase in fracture toughness was observed at 0.5 wt% reinforcement levels, with fracture toughness values ranging from 0.72 to 0.73 MPa$\sqrt{\text{m}}$.
— Incorporating nanomaterials using high speed and high shear followed by curing with an orbital shaker results in a 78% decrease in water absorption and a 76% decrease in porosity.
— Scratch tests offer an efficient and rigorous means to probe the size-independent fracture toughness of cement nanocomposites.

The protocols and findings reported in this study pave the way for discovering novel ways to increase the fraction of carbon-based nanomaterials within Portland cements to yield improvements in fracture toughness and water penetration resistance.

## Data accessibility

Scratch Test Data and Scanning Electron Microscopy Dryad Dataset: [40].
